# 4-Allyl-3-(2-methyl-4-quinol­yl)-1*H*-1,2,4-triazole-5(4*H*)-thione

**DOI:** 10.1107/S1600536810044697

**Published:** 2010-11-06

**Authors:** Artyom G. Kashaev, Anatoliy V. Zimichev, Victor B. Rybakov, Yurij N. Klimochkin, Margarita N. Zemtsova

**Affiliations:** a Samara State Technical University, Molodogvardeyskay Str. 244, 443100 Samara, Russian Federation; b Department of Chemistry, Moscow State University, 119992 Moscow, Russian Federation

## Abstract

In the title compound, C_15_H_14_N_4_S, the quinoline and triazole rings form a dihedral angle of 41.48 (7)°. In the crystal, adjacent mol­ecules are linked by N—H⋯N hydrogen bonds, forming chains along [100].

## Related literature

For the use of hydrazides and their functional derivatives in the preparation of a series of anti­tubercular and anti­bacterial compounds, see: Anghel & Silberg (1971 ▶); Figueiredo *et al.* (2000 ▶). 
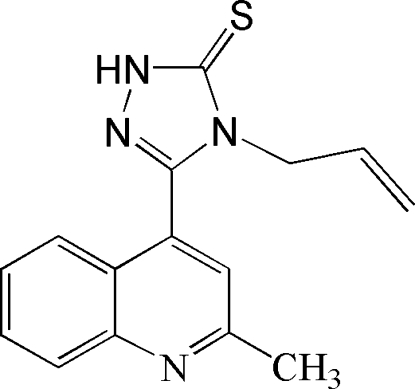


## Experimental

### 

#### Crystal data

C_15_H_14_N_4_S
*M* *_r_* = 282.37
Monoclinic, 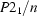

*a* = 7.8184 (8) Å
*b* = 11.5159 (13) Å
*c* = 15.7723 (14) Å
β = 96.034 (9)°
*V* = 1412.2 (3) Å^3^
*Z* = 4
Cu *K*α radiation
μ = 1.99 mm^−1^
*T* = 295 K
0.20 × 0.20 × 0.20 mm

#### Data collection

Enraf–Nonius CAD-4 diffractometer
2897 measured reflections
2897 independent reflections
2570 reflections with *I* > 2σ(*I*)
1 standard reflections every 60 min  intensity decay: 4%

#### Refinement

*R*[*F* ^2^ > 2σ(*F* ^2^)] = 0.061
*wR*(*F* ^2^) = 0.171
*S* = 1.09
2897 reflections
182 parameters
H-atom parameters constrained
Δρ_max_ = 0.28 e Å^−3^
Δρ_min_ = −0.60 e Å^−3^

### 

Data collection: *CAD-4 EXPRESS* (Enraf–Nonius, 1994 ▶); cell refinement: *CAD-4 EXPRESS*; data reduction: *XCAD4* (Harms & Wocadlo, 1995 ▶); program(s) used to solve structure: *SHELXS97* (Sheldrick, 2008 ▶); program(s) used to refine structure: *SHELXL97* (Sheldrick, 2008 ▶); molecular graphics: *ORTEP-3* (Farrugia, 1997 ▶); software used to prepare material for publication: *WinGX* (Farrugia, 1999 ▶).

## Supplementary Material

Crystal structure: contains datablocks global, I. DOI: 10.1107/S1600536810044697/ng5047sup1.cif

Structure factors: contains datablocks I. DOI: 10.1107/S1600536810044697/ng5047Isup2.hkl

Additional supplementary materials:  crystallographic information; 3D view; checkCIF report

## Figures and Tables

**Table 1:** Hydrogen-bond geometry (Å, °)

*D*—H⋯*A*	*D*—H	H⋯*A*	*D*⋯*A*	*D*—H⋯*A*
N14—H14⋯N1^i^	0.86	2.21	2.978 (3)	148

Symmetry code: (i) 
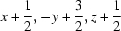
.

**Fig. 1.**
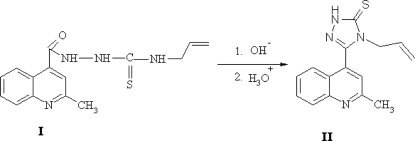
Synthesis of the title compound.

**Fig. 2.**
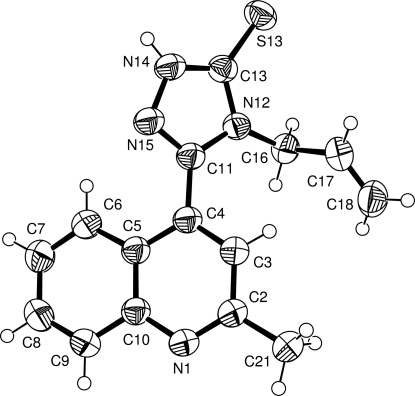
ORTEP–3 (Farrugia, 1997) plot of molecular structure of the title compound showing the atom–numbering scheme. Thermal displacement ellipsoids are drawn at the 50% probability level. H atoms are presented as a small spheres of arbitrary radius.

## supplementary crystallographic information

### Comment

Hydrazides and their functional derivatives were used to prepare a series of antitubercular and antibacterial compounds (Anghel & Silberg, 1971; Figueiredo *et al.*, 2000). Many heterocyclic compounds directly prepared from hydrazides, too, possess valuable biological and physicochemical properties. This causes an attention to new synthetic methods and investigation of similar compounds. *N*–(Allylthiocarbonyl)–4–(2–methyl–4–quinolyl)–carbohydrazide, I, was synthesized from allyl isothiocyanate and hydrazide 2–methyl–4–quinoline carboxylic acid. When heated with alkali for 1 h, the product undergoes cyclization into 4-allyl-3-(2-methyl-4-quinolyl)-4,5-dihydro-1*H*-1,2,4-triazole-5-thione, II (Fig. 1).

In title molecule, guinoline moiety is planar (max deviation of C6 = 0.039 (2) Å) and assential planar triazole moiety form dihedral angle 41.48 (7)° (Fig. 2). In the crystal structure is found classical hydrogen bond N14—H14···N1^i^ with parameters N14—H14 = 0.86 Å, N14···N1^i^ = 2.978 (3) Å, H14···N1^i^ = 2.21Å and angle N14—H14···N1^i^ = 147.8°. Non–classical H bond is found too: C21—H21A···N15^ii^ with parameters C21—H21A = 0.96 Å, C21···N15^ii^ = 3.330 (3) Å, H21A···N15^ii^ = 2.450Å and angle C21—H21A···N15^ii^ = 152°. Is found σ···π–interaction between H8—>C17^iii^═C18^iii^ (H8···C17^iii^ = 2.800Å and H8···C18^iii^ = 2.896 Å). Symmetry codes: (i) *x* + 1/2, -*y* + 3/2, *z* + 1/2; (ii) *x* - 1/2, -*y* + 3/2, *z* - 1/2; (iii) *x*, *y* - 1, *z*.

### Experimental

A solution of 1.43 mmol of NaOH in 10 ml of water was added to 0.95 mmol of *N*–(allylthiocarbonyl)–4–(2–methyl–4–quinolyl)–carbohydrazide, and the mixture was refluxed for 1 h. The solution was cooled and acidified with acetic acid to pH 4. The precipitate that formed was filtered off and recrystallized from ethanol. Recrystallization of the crude product from ethanol gave 0.2 g of colourless crystals. Yield 73%, m.p. 494–495 K.

IR, ν, cm^-1^: 3432 (NH), 3324 (NH), 1348 (C═S). MS, m/*z*: 282 (100) [*M*]+, 245 (39), 267 (62), 169 (66), 168 (61), 140 (23). ^1^H NMR, δ: 2.68 s (3*H*, CH_3_), 3.92 s (1*H*, NH), 4.53 d (2*H*, J = 5.04, —CH_2_—CH═CH_2_), 4.67 d (1*H*, J = 10.53, —CH═CH_2_*trans*), 5.17 dd (1*H*, J = 16.94, —CH═CH_2_*cis*), 5.62 m (1*H*, —CH═), 7.55 t (1*H*, J = 7.34, 7–H), 7.66 s (1*H*, 3–H), 7.76 t (1*H*, J = 7.74, 6–H), 7.78 d (1*H*, J = 8.24, 8–H), 8.00 d (1*H*, J = 8.70, 5–H). Anal. calc. for C_15_H_14_N_4_S, %: C 63.80; H 5.00; N 19.84; S 11.36. Found, %: C 63.68; H 5.09; N 11.44; S 11.31.

Single crystals for *X*–ray analysis were obtained by slow evaporation of an ethanol. IR spectrum was recorded (in KBr) on Shimadzu FTIR–8400S. Mass spectrum was measured on Finnigan Trance DSQ spectrometer. ^1^H NMR spectrum was obtained in *DMSO*–d_6_ on Bruker AM 300 (300 MHz), using *TMS* as internal standard. Elemental composition was determined on Euro Vector EA–3000 elemental analyzer.

### Refinement

C– or N–bound H–atoms were placed in calculated positions (C—H 0.93–0.97Å and N—H 0.86 Å) and refined as riding, with *U*_iso_(H) = 1.2(1.5)*U*_eq_(C, N).

### Crystal data

**Table:**

C_15_H_14_N_4_S	*F*(000) = 592
*M**_r_* = 282.37	*D*_x_ = 1.328 Mg m^−^^3^
Monoclinic, *P*2_1_/*n*	Melting point = 494–495 K
Hall symbol: -P 2yn	Cu *K*α radiation, λ = 1.54184 Å
*a* = 7.8184 (8) Å	Cell parameters from 25 reflections
*b* = 11.5159 (13) Å	θ = 29.9–32.4°
*c* = 15.7723 (14) Å	µ = 1.99 mm^−^^1^
β = 96.034 (9)°	*T* = 295 K
*V* = 1412.2 (3) Å^3^	Prism, colourless
*Z* = 4	0.20 × 0.20 × 0.20 mm

### Data collection

**Table:**

Enraf–Nonius CAD-4 diffractometer	*R*_int_ = 0.0000
Radiation source: Fine–focus sealed tube	θ_max_ = 74.9°, θ_min_ = 4.8°
Graphite	*h* = −9→9
non–profiled ω scans	*k* = 0→14
2897 measured reflections	*l* = 0→19
2897 independent reflections	1 standard reflections every 60 min
2570 reflections with *I* > 2σ(*I*)	intensity decay: 4%

### Refinement

**Table:**

Refinement on *F*^2^	Primary atom site location: structure-invariant direct methods
Least-squares matrix: full	Secondary atom site location: difference Fourier map
*R*[*F*^2^ > 2σ(*F*^2^)] = 0.061	Hydrogen site location: inferred from neighbouring sites
*wR*(*F*^2^) = 0.171	H-atom parameters constrained
*S* = 1.09	*w* = 1/[σ^2^(*F*_o_^2^) + (0.0953*P*)^2^ + 0.6742*P*] where *P* = (*F*_o_^2^ + 2*F*_c_^2^)/3
2897 reflections	(Δ/σ)_max_ = 0.001
182 parameters	Δρ_max_ = 0.28 e Å^−^^3^
0 restraints	Δρ_min_ = −0.60 e Å^−^^3^

### Special details

**Table:**

Geometry. All s.u.'s (except the e.s.d. in the dihedral angle between two l.s. planes) are estimated using the full covariance matrix. The cell s.u.'s are taken into account individually in the estimation of s.u.'s in distances, angles and torsion angles; correlations between s.u.'s in cell parameters are only used when they are defined by crystal symmetry. An approximate (isotropic) treatment of cell s.u.'s is used for estimating s.u.'s involving l.s. planes.
Refinement. Refinement of F^2^ against ALL reflections. The weighted R–factor wR and goodness of fit S are based on F^2^, conventional R–factors R are based on F, with F set to zero for negative F^2^. The threshold expression of F^2^ > 2σ(F^2^) is used only for calculating R–factors(gt) etc. and is not relevant to the choice of reflections for refinement. R–factors based on F^2^ are statistically about twice as large as those based on F, and R–factors based on ALL data will be even larger.

### Fractional atomic coordinates and isotropic or equivalent isotropic displacement parameters (Å^2^)

**Table:**

	*x*	*y*	*z*	*U*_iso_*/*U*_eq_	
N1	−0.0256 (3)	0.68265 (17)	0.64417 (12)	0.0489 (4)	
C2	0.0117 (3)	0.7945 (2)	0.64169 (14)	0.0510 (5)	
C21	−0.0078 (5)	0.8565 (2)	0.55821 (17)	0.0707 (8)	
H21A	−0.0327	0.8014	0.5129	0.106*	
H21B	0.0971	0.8967	0.5505	0.106*	
H21C	−0.1002	0.9115	0.5575	0.106*	
C3	0.0741 (3)	0.8566 (2)	0.71541 (14)	0.0504 (5)	
H3	0.1053	0.9340	0.7105	0.060*	
C4	0.0898 (3)	0.80569 (19)	0.79389 (13)	0.0450 (5)	
C5	0.0448 (3)	0.6859 (2)	0.79967 (13)	0.0452 (5)	
C6	0.0506 (4)	0.6230 (2)	0.87675 (16)	0.0572 (6)	
H6	0.0809	0.6605	0.9284	0.069*	
C7	0.0115 (4)	0.5067 (2)	0.87563 (18)	0.0677 (7)	
H7	0.0161	0.4656	0.9266	0.081*	
C8	−0.0351 (4)	0.4498 (2)	0.79841 (18)	0.0636 (7)	
H8	−0.0601	0.3708	0.7986	0.076*	
C9	−0.0446 (3)	0.5078 (2)	0.72313 (15)	0.0521 (5)	
H9	−0.0757	0.4685	0.6723	0.063*	
C10	−0.0073 (3)	0.62756 (19)	0.72191 (14)	0.0445 (5)	
C11	0.1589 (3)	0.87239 (19)	0.86904 (13)	0.0438 (5)	
N12	0.1212 (2)	0.98612 (16)	0.88595 (11)	0.0426 (4)	
C13	0.2140 (3)	1.0161 (2)	0.96191 (13)	0.0461 (5)	
S13	0.20807 (9)	1.14068 (5)	1.01518 (4)	0.0584 (2)	
N14	0.3062 (3)	0.91993 (18)	0.98323 (11)	0.0509 (5)	
H14	0.3792	0.9153	1.0279	0.061*	
N15	0.2733 (3)	0.83085 (18)	0.92762 (12)	0.0512 (5)	
C16	−0.0166 (3)	1.0582 (2)	0.84356 (15)	0.0479 (5)	
H16A	−0.0686	1.1025	0.8864	0.057*	
H16B	−0.1045	1.0080	0.8154	0.057*	
C17	0.0426 (3)	1.1402 (2)	0.77948 (17)	0.0544 (6)	
H17	0.1430	1.1819	0.7946	0.065*	
C18	−0.0369 (4)	1.1571 (3)	0.70368 (19)	0.0670 (7)	
H18A	−0.1377	1.1167	0.6865	0.080*	
H18B	0.0070	1.2096	0.6667	0.080*	

### Atomic displacement parameters (Å^2^)

**Table:**

	*U*^11^	*U*^22^	*U*^33^	*U*^12^	*U*^13^	*U*^23^
N1	0.0549 (11)	0.0500 (10)	0.0402 (9)	0.0018 (8)	−0.0018 (8)	−0.0032 (8)
C2	0.0626 (14)	0.0476 (12)	0.0413 (11)	0.0030 (10)	−0.0012 (9)	−0.0011 (9)
C21	0.109 (2)	0.0540 (15)	0.0459 (13)	−0.0009 (14)	−0.0077 (14)	0.0031 (11)
C3	0.0633 (14)	0.0436 (12)	0.0431 (12)	0.0006 (10)	−0.0004 (10)	−0.0017 (9)
C4	0.0485 (11)	0.0460 (11)	0.0398 (11)	0.0019 (9)	0.0016 (8)	−0.0034 (9)
C5	0.0489 (11)	0.0448 (11)	0.0413 (11)	0.0035 (9)	0.0025 (8)	−0.0019 (9)
C6	0.0772 (17)	0.0522 (13)	0.0417 (12)	−0.0016 (12)	0.0037 (11)	0.0006 (10)
C7	0.099 (2)	0.0514 (14)	0.0521 (14)	−0.0034 (14)	0.0051 (13)	0.0072 (11)
C8	0.0819 (19)	0.0430 (12)	0.0656 (16)	−0.0012 (12)	0.0059 (13)	−0.0003 (11)
C9	0.0570 (13)	0.0467 (12)	0.0517 (13)	0.0013 (10)	0.0017 (10)	−0.0068 (10)
C10	0.0457 (11)	0.0454 (11)	0.0421 (11)	0.0032 (9)	0.0027 (8)	−0.0024 (8)
C11	0.0487 (11)	0.0439 (11)	0.0384 (10)	0.0003 (9)	0.0022 (8)	−0.0025 (8)
N12	0.0456 (9)	0.0429 (9)	0.0392 (9)	−0.0006 (7)	0.0045 (7)	−0.0013 (7)
C13	0.0497 (11)	0.0497 (12)	0.0394 (10)	−0.0056 (9)	0.0070 (8)	−0.0023 (9)
S13	0.0753 (5)	0.0485 (4)	0.0524 (4)	−0.0073 (3)	0.0107 (3)	−0.0104 (2)
N14	0.0568 (11)	0.0537 (11)	0.0402 (9)	0.0034 (9)	−0.0039 (8)	−0.0066 (8)
N15	0.0582 (11)	0.0496 (10)	0.0436 (10)	0.0068 (9)	−0.0044 (8)	−0.0062 (8)
C16	0.0475 (11)	0.0469 (12)	0.0498 (12)	0.0049 (9)	0.0071 (9)	0.0032 (9)
C17	0.0574 (13)	0.0440 (12)	0.0626 (14)	−0.0004 (10)	0.0106 (11)	0.0053 (10)
C18	0.0805 (19)	0.0596 (15)	0.0617 (16)	0.0015 (13)	0.0115 (13)	0.0118 (12)

### Geometric parameters (Å, °)

**Table:**

N1—C2	1.322 (3)	C8—H8	0.9300
N1—C10	1.375 (3)	C9—C10	1.411 (3)
C2—C3	1.408 (3)	C9—H9	0.9300
C2—C21	1.492 (3)	C11—N15	1.307 (3)
C21—H21A	0.9600	C11—N12	1.375 (3)
C21—H21B	0.9600	N12—C13	1.377 (3)
C21—H21C	0.9600	N12—C16	1.465 (3)
C3—C4	1.363 (3)	C13—N14	1.345 (3)
C3—H3	0.9300	C13—S13	1.666 (2)
C4—C5	1.430 (3)	N14—N15	1.357 (3)
C4—C11	1.467 (3)	N14—H14	0.8600
C5—C6	1.411 (3)	C16—C17	1.491 (3)
C5—C10	1.420 (3)	C16—H16A	0.9700
C6—C7	1.374 (4)	C16—H16B	0.9700
C6—H6	0.9300	C17—C18	1.303 (4)
C7—C8	1.397 (4)	C17—H17	0.9300
C7—H7	0.9300	C18—H18A	0.9300
C8—C9	1.357 (4)	C18—H18B	0.9300
			
C2—N1—C10	118.25 (19)	C10—C9—H9	120.0
N1—C2—C3	121.9 (2)	N1—C10—C9	117.5 (2)
N1—C2—C21	119.4 (2)	N1—C10—C5	123.1 (2)
C3—C2—C21	118.7 (2)	C9—C10—C5	119.4 (2)
C2—C21—H21A	109.5	N15—C11—N12	110.86 (19)
C2—C21—H21B	109.5	N15—C11—C4	123.1 (2)
H21A—C21—H21B	109.5	N12—C11—C4	125.98 (19)
C2—C21—H21C	109.5	C11—N12—C13	107.68 (18)
H21A—C21—H21C	109.5	C11—N12—C16	127.87 (18)
H21B—C21—H21C	109.5	C13—N12—C16	123.32 (19)
C4—C3—C2	121.5 (2)	N14—C13—N12	103.32 (19)
C4—C3—H3	119.3	N14—C13—S13	128.73 (17)
C2—C3—H3	119.3	N12—C13—S13	127.93 (18)
C3—C4—C5	118.3 (2)	C13—N14—N15	113.62 (18)
C3—C4—C11	119.9 (2)	C13—N14—H14	123.2
C5—C4—C11	121.75 (19)	N15—N14—H14	123.2
C6—C5—C10	118.8 (2)	C11—N15—N14	104.45 (19)
C6—C5—C4	124.3 (2)	N12—C16—C17	113.73 (19)
C10—C5—C4	116.8 (2)	N12—C16—H16A	108.8
C7—C6—C5	120.1 (2)	C17—C16—H16A	108.8
C7—C6—H6	119.9	N12—C16—H16B	108.8
C5—C6—H6	119.9	C17—C16—H16B	108.8
C6—C7—C8	120.4 (2)	H16A—C16—H16B	107.7
C6—C7—H7	119.8	C18—C17—C16	124.4 (3)
C8—C7—H7	119.8	C18—C17—H17	117.8
C9—C8—C7	121.1 (2)	C16—C17—H17	117.8
C9—C8—H8	119.4	C17—C18—H18A	120.0
C7—C8—H8	119.4	C17—C18—H18B	120.0
C8—C9—C10	120.0 (2)	H18A—C18—H18B	120.0
C8—C9—H9	120.0		
			
C10—N1—C2—C3	2.0 (4)	C4—C5—C10—C9	176.7 (2)
C10—N1—C2—C21	−179.7 (2)	C3—C4—C11—N15	−135.3 (3)
N1—C2—C3—C4	−3.8 (4)	C5—C4—C11—N15	41.9 (3)
C21—C2—C3—C4	178.0 (3)	C3—C4—C11—N12	41.3 (3)
C2—C3—C4—C5	1.4 (4)	C5—C4—C11—N12	−141.6 (2)
C2—C3—C4—C11	178.6 (2)	N15—C11—N12—C13	−2.3 (3)
C3—C4—C5—C6	−178.3 (2)	C4—C11—N12—C13	−179.2 (2)
C11—C4—C5—C6	4.5 (4)	N15—C11—N12—C16	−170.3 (2)
C3—C4—C5—C10	2.3 (3)	C4—C11—N12—C16	12.7 (4)
C11—C4—C5—C10	−174.9 (2)	C11—N12—C13—N14	2.6 (2)
C10—C5—C6—C7	2.0 (4)	C16—N12—C13—N14	171.29 (19)
C4—C5—C6—C7	−177.3 (3)	C11—N12—C13—S13	−175.96 (17)
C5—C6—C7—C8	−0.3 (5)	C16—N12—C13—S13	−7.2 (3)
C6—C7—C8—C9	−0.7 (5)	N12—C13—N14—N15	−2.2 (3)
C7—C8—C9—C10	−0.1 (4)	S13—C13—N14—N15	176.36 (17)
C2—N1—C10—C9	−178.8 (2)	N12—C11—N15—N14	0.9 (3)
C2—N1—C10—C5	2.0 (3)	C4—C11—N15—N14	178.0 (2)
C8—C9—C10—N1	−177.5 (2)	C13—N14—N15—C11	0.8 (3)
C8—C9—C10—C5	1.8 (4)	C11—N12—C16—C17	−100.9 (3)
C6—C5—C10—N1	176.5 (2)	C13—N12—C16—C17	92.8 (3)
C4—C5—C10—N1	−4.1 (3)	N12—C16—C17—C18	134.9 (3)
C6—C5—C10—C9	−2.7 (3)		

### Hydrogen-bond geometry (Å, °)

**Table:**

*D*—H···*A*	*D*—H	H···*A*	*D*···*A*	*D*—H···*A*
N14—H14···N1^i^	0.86	2.21	2.978 (3)	148

Symmetry codes: (i) *x*+1/2, −*y*+3/2, *z*+1/2.
